# Self-Reported Level of Cultural Competence of Midwives in the North-West Province of South Africa

**DOI:** 10.1177/10436596231175165

**Published:** 2023-06-05

**Authors:** Khumoetsile Daphney Shopo, Antoinette Du Preez, Tinda Rabie, Petra Bester

**Affiliations:** 1 North–West University, Potchefstroom, South Africa

**Keywords:** cultural competence, midwives, Transcultural Self-Efficacy Tool, South Africa

## Abstract

**Introduction:**

Limited research on cultural competence in nursing, midwifery, and education exists within low- and middle-income countries such as South Africa (SA). This study aimed to describe midwives’ self-reported levels of cultural competence toward women receiving maternal care.

**Methodology:**

A descriptive, cross-sectional survey design and an all-inclusive sample of (*N* = 104; *n* = 82) midwives yielded a 79% response rate. Data were collected using the Transcultural Self-Efficacy Tool (TSET) questionnaire. Participants included midwives recruited from maternity units of five hospitals in South Africa: different hospitals that included one large district, two regional, and two tertiary hospitals in the North-West Province of SA. An all-inclusive sample of (*N* = 104; *n* = 82) midwives participated, representing a 79% response rate.

**Results:**

Midwives reported an overall moderate level of competence regarding their knowledge and understanding of cultural factors; it was concerning that their confidence in interviewing patients from different cultural backgrounds on factors such as acculturation and worldview were the lowest.

**Discussion:**

To the best of the authors’ knowledge, this was the first study to assess the cultural competence of midwives in SA using TSET. The study highlighted the need for midwives’ training to improve their cultural competence.

## Introduction

Increased cultural competence of nurses and midwives has the potential to improve patient’s satisfaction levels and health outcomes. Research emphasized that health professionals who lack cultural competence may contribute to delays in patient treatment, non-compliance with treatment, and inappropriate diagnoses (De Beer & Chipps, 2014). Madeline Leininger was the first nurse to venture into transcultural nursing care in the early 1980s, and she developed a theory to improve and provide culturally competent care to patients (McFarland, 2006). Culture is referred to as “patterned lifeways, values, beliefs, norms, symbols, and practices of individuals, groups, or institutions that are learned, shared, and usually transmitted from one generation to another” (Leininger *in* McFarland, 2006). However, it is essential to note that people from the same race will not always share the same cultural beliefs and/or practices, with specific reference to maternal health beliefs and practices.

Cultural competence in nursing, midwifery, and education has been researched extensively in high-income countries, such as the Americas and Europe, to address racial and ethnic inequities (Prosen, 2015; Shen, 2015; Vaughn, 2008). Cultural competence, according to Campinha-Bacote (2002), is the ongoing process in which the health care provider continually strives to achieve the ability to work within the cultural context of the patient effectively. That process involves the integration of five constructs, namely—(a) cultural awareness, (b) cultural knowledge, (c) cultural skill, (d) cultural encounters, and (e) cultural desire. Therefore, to provide culturally competent care, midwives who are the focus of this study should exhibit specific skills, knowledge, and competencies.

De Beer and Chipps (2014) argued that it is essential for health care workers (midwives in this study) to integrate culture and language into their care as this ultimately improves health outcomes of patients from diverse cultural backgrounds. However, the authors identified the need for more literature relating to the cultural competence of midwives within the South African (SA) context. A recent qualitative study found that the midwives in the North-West Province (NWP) of SA lacked cultural knowledge and insights into the maternal patient’s cultural practices (Shopo et al., 2023). The objective of the current study was to describe midwives’ self-reported levels of cultural competence toward women receiving maternal care using a Transcultural Self-Efficacy Tool (TSET).

### Methods

A descriptive, cross-sectional survey design (Gray et al., 2017) was used in this study. The NWP has 19 hospitals divided into seven small, four medium, one large, three regional, two tertiary, and two specialized psychiatric hospitals. Five hospital maternity units were purposively selected as they provided rich culturally diverse participants to inform the study. Participants included midwives recruited from maternity units of five different hospitals that included one large district, two regional, and two tertiary hospitals in a province of SA. An all-inclusive sample of (*N* = 104; *n* = 82) midwives participated, representing a 79% response rate. The number of midwives in the five selected hospitals confirms the shortage of midwives in the country. Inclusion criteria included permanently employed midwives working in one of the five identified maternity units in the NWP, having more than 1 year of experience, and being able to read and communicate in English.

### Instrument

The TSET questionnaire by Jeffreys (2010), measuring cultural competence, was used in this study. No tool is available in SA to measure the cultural competency of nurses or midwives; hence, permission was sought from the publisher to use TSET. One question in the 83-item questionnaire was slightly adapted to suit the South African context with permission from the developer. Item 71 was changed from “Inadequacies in *the US health care system*” to “Inadequacies *in the SA health care system.*”

The original demographic section was not included as it was not applicable to the context of this study; only age, home language, and highest qualification of participants were included. The original TSET is an 83-item questionnaire divided into three parts, namely Part I (Cognitive aspects—25 items), Part II (Practical aspects—28 items), and Part III (Affective aspects—30 items). In Part I, the nurses rated themselves on confidence levels relating to their knowledge and understanding of how cultural factors may influence nursing care among patients of different cultural backgrounds. Part II included midwives’ confidence rating when interviewing patients from different cultural backgrounds, including their values and beliefs. Part III assessed the nurses’ knowledge of themselves in terms of cultural awareness and that of different patients, the acceptance of similarities and differences, appreciation, recognition, and advocacy regarding client’s decisions and culture-specific care (Jeffreys & Dogan, 2010).

### Data Collection

After ethical clearance from relevant authorities, data collection commenced. The Health Research Ethics Committee (HREC) of the North-West University (NWU) granted ethical clearance for the study (Ethics number: NWU-00067-18-S1). The authors sought the assistance of independent persons who were Administrative Clerks employed at the five hospitals to assist with recruitment. Permission was given by the North West Provincial Department of Health to access the hospitals included in the study. Thereafter, permission was obtained from the management of each hospital and, thereafter, the unit managers of the maternity units. Recruitment was done by placing posters in maternity units, with the assistance of independent persons. The participants interested and willing to participate signed informed consent forms. Participants were then issued with TSET questionnaires. Clear instructions were provided to participants to place the completed questionnaires in a sealed box in each maternity unit and were later collected.

### Data Analysis

Data analysis was conducted with the assistance of a statistician at the university. Statistical Package for Social Services (SPSS) version 25 was used for data analysis and included descriptive and exploratory factors analysis in determining frequency distribution of variables through mean, percentages, and standard deviation (*SD*). A two-tailed test with significance level of .05 was used to interpret the findings. The percentage of variance explained is consistently above 50%; in this questionnaire, Part I = 60.94%; Part II = 58.0%, and Part III = 79.29%. The Kaiser–Meyer–Olkin (KMO) test of sampling adequacy measured the variance of the items on the questionnaire (Friel, cited in Beavers et al., 2013). The KMO test for Part I (knowledge and understanding) of the questionnaire with 25 items was .878, Part II (interviewing) with 28 items was .885, and finally, Part III (self and patients’ awareness) had 30 items scored .871.

Oblimin rotation was used to maximize high item loadings and minimize low item loadings. The factorability of the loadings derives from the pattern matrix and should be above 0.3. Williams et al. (2010, p. 9) explained that the factorability of 0.3 indicates that factors account for about 30% of relationship within the data. Table 1 depicts the pattern matrix for the affective subscale.

**Table 1.** Pattern Matrix for Affective Subscales.

Item no.	Item	Component					
		1	2	3	4	5	6
Factor 1: Self-awareness							
54	Your own cultural heritage and belief systems					0.797	
55	Your own biases and limitations					0.741	
56	Differences within your own cultural group					0.699	
Factor 2: Patients’ awareness							
57	Insensitive and prejudicial treatment	0.750					
58	Differences in perceived role of the nurse	0.850					
59	Traditional caring behaviors	0.838					
60	Professional caring behaviors	0.990					
61	Comfort and discomfort felt when entering a culturally different world	0.788					
62	Interaction between nursing, indigenous, and professional systems	0.698					
Factor 3: Acceptance							
63	Differences between cultural groups				0.407		
64	Similarities between cultural groups				0.477		
65	Patient’s refusal of treatment based on beliefs				−0.318		
Factor 4: Appreciate							
66	Interaction with people of different cultures				0.341		
67	Cultural sensitivity and awareness				0.570		
68	Culture-specific nursing care				0.598		
69	Role of family in providing health care				0.929		
70	Patient’s worldview (philosophy of life)				0.867		
Factor 5: Recognize							
71	Inadequacies in the SA health care system		−0.704				
72	Importance of home remedies and indigenous medicine		−0.827				
73	Impact of roles on health care practices		−0.730				
74	Impact of values on health care practices		−0.729				
75	Impact of socioeconomic factors on health care practices		−0.826				
76	Impact of political factors on health care practices		−0.760				
77	Need for cultural care preservation/maintenance		−0.567				
78	Need for cultural care accommodation/negotiation		−0.405				
79	Need for cultural care repatterning/restructuring		−0.389				
80	Need to prevent ethnocentric views		−0.331				
81	Need to prevent cultural imposition		−0.343				
Factor 6: Advocate							
82	Patient’s decisions based on cultural beliefs			−0.860			
83	Culture-specific care			−0.922			

### Ethical Considerations

The ethical clearance for the study was granted by HREC of the NWU. Further permission was sought from the Provincial Department of Health to gain access to hospitals. All participants signed an informed consent form before completing the questionnaires. The confidentiality of participants was maintained. Participants did not have to write their names or any identifying information on the consent form or the questionnaire. The information provided in the questionnaire cannot be linked to participants in no way.

## Results

### Demographic Data of Participants

The majority of respondents were in the age group 40 to 49 years (*n* = 33; 40.2%), followed by 50 years and above age group with 21 respondents (25.6%), 30 to 39 years was 19 (23.2%), while the least representation of six respondents was the age group 20 to 29 years (7.3%). The mean age of respondents was 25 years, similar to the study by Jin and Cleofas (2018), where the participants were 21 to 29 years old. Regarding qualification level, majority of respondents (*n* = 31; 37.8%) had the Diploma qualification, followed by 27 respondents (32.9%) who had Advanced Diploma qualifications, only 19 (23.2%) respondents had a degree, and three respondents (3.7%) had a postgraduate qualification, either a Master’s or a PhD. The language aspect of the demographics indicated the majority of respondents (*n* = 53; 64.6%) spoke Setswana, followed by nine who spoke IsiXhosa (11.0%), four who spoke English (4.9%), and three who spoke Afrikaans (3.7%), while (*n* = 8; 9.8%) represented other languages of 11 official languages in SA.

### Descriptive Statistics

Descriptive statistics were discussed according to different parts of the questionnaire: Part I named the cognitive subscale, Part II named practical subscale, and Part III named affective subscale.

#### Part I (Cognitive Subscale)

The mean scoring is from 1 to 10, a score closer to 10 indicating a higher self-efficacy level in their knowledge and understanding of listed items. The midwives perceived themselves as on the moderate level of cultural competence because their confidence levels were closer to 10 on the Likert-type scale. Table 2 depicts Part I items with each item’s resulting means and *SD*.

**Table 2.** Part I Items, Means, and Standard Deviations.

Item numbers	Areas of nursing care that might be affected by cultural factors	*M*	*SD*
1	Health history and interview	7.28	1.98
2	Physical examination	7.74	1.82
3	Informed consent	7.90	1.84
4	Health promotion	7.55	1.95
5	Illness prevention	7.30	1.98
6	Health maintenance	7.24	1.88
7	Health restoration	6.89	2.16
8	Safety	7.12	2.30
9	Exercise and activity	7.43	1.98
10	Pain relief and comfort	7.83	2.03
11	Diet and nutrition	7.34	2.06
12	Patient teaching	7.82	2.07
13	Hygiene	7.53	2.11
14	Anxiety and stress reduction	7.18	2.06
15	Diagnostic tests	6.93	2.25
16	Blood tests	7.09	2.30
17	Pregnancy	7.72	2.08
18	Birth	7.87	1.88
19	Growth and development	7.40	1.95
20	Aging	7.15	2.01
21	Dying and death	6.99	2.07
22	Grieving and loss	7.25	2.15
23	Life support and resuscitation	7.33	2.21
24	Sexuality	6.69	2.24
25	Rest and sleep	7.22	2.48
Overall mean		7.36	1.61

Midwives felt confident in knowing and understanding how obtaining informed consent from patients can be influenced by cultural factors. This is evidenced by the highest mean score of 7.90 (*SD* = 1.84), closer to 10 (highest confidence level). The second highest mean score of 7.87 (*SD* = 1.88) confirms that midwives know and understand how cultural factors may influence their care regarding birth. This result is significant as this study focuses on the cultural competence of midwives in rendering culturally congruent maternal care.

Midwives have a lower confidence level on health restoration, with a mean score of 6.89 (*SD* = 2.16); diagnostic tests mean score of 6.93 (*SD* = 2.25); and cultural factors can influence death and dying, with a mean score of 6.99 (*SD* = 2.07). These mean scores indicate that most participants had some confidence in terms of the Cognitive items listed and their influence on nursing care, in this instance, maternal care; this is indicated by a moderate overall mean score of 7.36 (*SD* = 1.61).

#### Part II (Practical Subscale)

Part II, as depicted in Table 3 indicated that midwives showed the highest confidence in “interviewing” patients about their religious background and identity, with a mean score of 7.69 (*SD* = 1.74). This is followed by a mean score of 7.66 (*SD* = 1.81) relating to their confidence level when interviewing patients about the role of family during illness. The lowest mean score of 5.91 (*SD* = 2.24) is related to interviewing about acculturation. Midwives are expected to be competent in interviewing patients in all aspects of care to be able to provide culturally congruent maternal care. It is concerning that most items in this subscale (21 out of 28 items) had a mean score below point 7 on the Likert-type scale. Generally, the mean score of 6.88 (*SD* = 1.63) indicates that midwives rated Practical subscale moderate. Table 3 below depicts the results on Prcatical subscale.

**Table 3.** Part II Items, Mean, and Standard Deviations.

Item numbers	Interview topics	*M*	*SD*
26	Language preference	6.96	2.34
27	Level of English comprehension	7.46	2.07
28	Meaning of verbal communication patterns	7.09	2.09
29	Meaning of nonverbal behaviors	6.43	2.21
30	Meanings of space and touch	6.75	2.11
31	Time perception and orientation	6.89	2.12
32	Racial background and identity	7.02	2.13
33	Ethnic background and identity	6.89	2.07
34	Socioeconomic background	7.18	1.88
35	Religious background and identity	7.69	1.74
36	Educational background and interests	7.33	2.10
37	Religious practices and beliefs	7.38	2.04
38	Acculturation	5.91	2.24
39	Worldview (philosophy of life)	6.15	2.28
40	Attitudes about health care technology	6.85	2.26
41	Ethnic food preferences	6.72	2.26
42	Role of elders	6.78	2.32
43	Role of children	6.71	2.24
44	Financial concerns	6.59	2.26
45	Traditional health and illness beliefs	6.56	2.23
46	Folk medicine tradition and use	6.40	2.41
47	Gender role and responsibility	6.98	2.19
48	Acceptable sick role behaviors	6.89	2.18
49	Role of family during illness	7.66	1.81
50	Discrimination and bias experiences	6.64	2.35
51	Home environment	6.93	2.18
52	Relationship ties	6.88	2.08
53	Aging	6.88	2.17
Overall mean		6.88	1.63

#### Part III (Affective Subscale)

Part III is made up of six factors where midwives rated themselves on their degree of confidence in terms of their self-awareness (items 54–56), patients’ awareness (items 57–62), acceptance (items 63–65), appreciation (items 66–70), recognition (items 71–81), and advocacy (items 82–83) for patients from different cultural backgrounds. Table 4 depicts the mean and *SD* results for different aspects in the affective subscale.

**Table 4:** Part III Subscale With Mean and Standard Deviations of Different Aspects.

Item numbers	Items on the affective subscale	*M*	*SD*
Section A: Self-awareness of the midwife			
Factor 1: Self-awareness			
54	Your own cultural heritage and belief systems	8.50	1.90
55	Your own biases and limitations	8.29	1.55
56	Differences within your own cultural group	8.44	1.38
Overall mean for Factor 1		8.41	1.36
Section B: Among patients of different cultural backgrounds			
Factor 2: Patients’ awareness			
57	Insensitive and prejudicial treatment	7.36	2.20
58	Differences in perceived role of the nurse	7.39	2.00
59	Traditional caring behaviors	6.87	2.25
60	Professional caring behaviors	7.82	2.01
61	Comfort and discomfort felt when entering a culturally different world	7.22	2.09
62	Interaction between nursing, indigenous, and professional systems	7.09	2.15
Overall mean for Factor 2		7.28	1.86
Factor 3: Acceptance			
63	Differences between cultural groups	7.98	1.71
64	Similarities between cultural groups	7.89	1.77
65	Patient’s refusal of treatment based on beliefs	6.96	2.37
Overall mean for Factor 3		7.60	1.62
Factor 4: Appreciation			
66	Interaction with people of different cultures	8.09	1.83
67	Cultural sensitivity and awareness	7.84	1.87
68	Culture-specific nursing care	7.35	2.12
69	Role of family in providing health care	7.93	1.86
70	Patient’s worldview (philosophy of life)	7.34	2.13
Overall mean for Factor 4		7.71	1.71
Factor 5: Recognition			
71	Inadequacies in the SA health care system	7.56	2.05
72	Importance of home remedies and indigenous medicine	6.43	2.32
73	Impact of roles on health care practices	7.16	1.89
74	Impact of values on health care practices	7.17	1.89
75	Impact of socioeconomic factors on health care practices	7.46	1.98
76	Impact of political factors on health care practices	7.38	2.06
77	Need for cultural care preservation/maintenance	6.74	2.13
78	Need for cultural care accommodation/negotiation	6.93	2.09
79	Need for cultural care repatterning/restructuring	6.67	1.96
80	Need to prevent ethnocentric views	6.76	2.09
81	Need to prevent cultural imposition	7.05	2.24
Overall mean for Factor 5		7.02	1.64
Factor 6: Advocate			
82	Patient’s decisions based on cultural beliefs	7.26	2.17
83	Culture-specific care	6.94	2.18
Overall mean for Factor 6		7.09	2.11

Highest confidence level is recorded for the midwives’ awareness about their own cultural heritage and belief systems on Factor 1 with a mean score of 8.50 (*SD* = 1.90), followed by the awareness of differences of own cultural group (8.44; *SD* = 1.38) and on their own biases and limitations (8.29; *SD =* 1.55), respectively. This rating should be higher because midwives are reflecting on their self-awareness perception. Furthermore, item 66, relating to midwives’ awareness of patients of different cultural backgrounds, related to the appreciation of interaction with people of different cultures and had a mean score of 8.09 (*SD* = 1.83), indicating higher confidence levels. Lowest mean scores on this subscale were Factor 5; item 72, relating to midwives’ recognition of the importance of home remedies and indigenous medicine among patients of different cultural backgrounds, had a mean score of 6.43 (*SD* = 2.32), and item 79, relating to cultural care repatterning/restructuring, with a mean score of 6.67 (*SD* = 1.96). Factor 6, item 83 focusing on the culture-specific care responsibilities of the midwives to advocate for patients, also had a low mean score of 6.94 (*SD* = 2.18).

### Correlation Between the Factors and Demographic Data of Midwives

There was no correlation between the qualifications and midwives’ perceptions of their self-efficacy. However, age positively correlates with the items on the TSET questionnaire. There was a positive correlation of .429 with the age of the midwives and Part I. This means an increase in the age of midwives is associated with an increase in their confidence level on their understanding and knowledge of cultural factors that may affect their maternal care. Table 5 below depicts the descriptive statistics on correlation between factors on the questionnaire and demographic data.

**Table 5.** Descriptive Statistics on Correlation Between Factors and Demographic Data.

Items		Age	Qualification
Part I (Knowledge and understanding)	Correlation coefficient	.429**	0.008
	Sig. (two-tailed)	0.000	0.945
	*N*	79	80
Part II (Interviewing)	Correlation coefficient	0.163	−0.057
	Sig. (two-tailed)	0.152	0.616
	*N*	79	80
Part III (Self- and patients’ awareness)			
Factor 1: Self-awareness	Correlation coefficient	0.105	−0.120
	Sig. (two-tailed)	0.357	0.290
	*N*	79	80
Factor 2: Patients’ awareness	Correlation coefficient	.230*	−0.116
	Sig. (two-tailed)	0.041	0.306
	*N*	79	80
Factor 3: Acceptance	Correlation coefficient	.069	−0.029
	Sig. (two-tailed)	0.544	0.801
	*N*	79	80
Factor 4: Appreciate	Correlation coefficient	.074	0.046
	Sig. (two-tailed)	0.518	0.687
	*N*	79	80
Factor 5: Recognize	Correlation coefficient	.033	0.025
	Sig. (two-tailed)	0.773	0.829
	*N*	79	80
Factor 6: Advocate	Correlation coefficient	.227*	0.063
	Sig. (two-tailed)	0.044	0.580
	*N*	79	80

* Correlation is significant at the .05 level (two-tailed).

** Correlations significant at the .01 level (two-tailed).

Another positive correlation is reported in Part III, whereby age positively correlates with Factor 2, named “patients’ awareness” (.230) and Factor 6, named “advocate” (.227), respectively. This indicated that an increase in the age of the midwives results in an increase in self-efficacy perception levels about their awareness of different items, such as insensitive and prejudicial treatment (item 57), among patients of different cultural backgrounds, differences in the perceived role of the nurse (item 58) and interaction between nursing, indigenous, and professional systems (item 62). These results also indicate that an increase in the age of the midwives is associated with their increase in self-efficacy perception levels on their advocacy for patients’ decisions based on cultural beliefs and culture-specific care.

### ANOVA on Hospitals

For the hospitals, one-way analysis of variance (ANOVA; Botma et al., 2010) measured the correlation with factors. The ANOVA revealed a statistically significant difference between the hospitals and Part I. However, according to Ellis and Steyn (2003), statistical significance does not necessarily indicate the result is vital in practice. Practical significance (effect sizes = *d*) is explained as a large enough difference to have an effect in practice. This measure was introduced by Ellis and Steyn (2003), who delineated the interpretation of results as follows: (small effect: *d =* 0.2; medium effect: *d* = 0.5; large effect: *d* = 0.8). Correlation data with an effect size of 0.8 is therefore practically significant (Ellis & Steyn, 2003). Practical significance is more appropriate as the sampling method for this study was all-inclusive. Table 6 depicts the descriptive statistics on perceptions of midwives grouped according to hospitals.

**Table 6:** Descriptive Statistics on Midwives’ Perception on Factors.

Items	Hospitals	*n*	*M*	*SD*	*p*-value	Effect sizes (*d*)			
						Hospital 1 with	Hospital 2 with	Hospital 3 with	Hospital 4 with
Part I (Knowledge and understanding)	1	17	7.92	1.51					
	2	13	8.38	1.24		0.30			
	3	11	5.78	1.75		1.23	1.49		
	4	15	7.18	1.53		0.49	0.78	0.80	
	5	26	7.26	1.35		0.44	0.83	0.85	0.05
	Total (*N*)	82	7.36	1.62	0.001				
Part II (Interviewing)	1	17	6.88	1.57					
	2	13	7.05	1.74		0.10			
	3	11	6.32	1.98		0.29	0.37		
	4	15	7.18	1.41		0.19	0.07	0.44	
	5	26	6.88	1.66		0.00	0.10	0.28	0.18
	Total (*N*)	82	6.89	1.64	0.756				
Part III (Self- and patients’ awareness)									
Factor 1: Self-awareness	1	17	7.88	1.70					
	2	13	7.97	1.62		0.05			
	3	11	8.39	1.32		0.30	0.26		
	4	15	8.71	1.00		0.49	0.45	0.24	
	5	26	8.81	1.08		0.55	0.51	0.31	0.09
	Total (*N*)	82	8.41	1.37	0.145				
Factor 2: Patients’ awareness	1	17	6.95	2.01					
	2	13	7.12	2.46		0.07			
	3	11	6.86	1.89		0.04	0.11		
	4	15	7.41	1.83		0.23	0.12	0.29	
	5	26	7.71	1.45		0.37	0.24	0.45	0.16
	Total (*N*)	82	7.29	1.87	0.638				
Factor 3: Acceptance	1	17	7.16	1.73					
	2	13	7.31	2.01		0.07			
	3	11	7.30	1.44		0.08	0.00		
	4	15	8.51	1.08		0.78	0.60	0.84	
	5	26	7.67	1.59		0.29	0.18	0.23	0.53
	Total (*N*)	82	7.61	1.63	0.146				
Factor 4: Appreciate	1	17	7.41	1.87					
	2	13	8.06	1.91		0.34			
	3	11	7.62	1.76		0.11	0.23		
	4	15	8.17	1.25		0.41	0.06	0.32	
	5	26	7.53	1.77		0.06	0.28	0.05	0.37
	Total (*N*)	82	7.72	1.72	0.658				
Factor 5: Recognize	1	17	6.84	1.28					
	2	13	6.50	2.53		0.14			
	3	11	7.07	1.35		0.17	0.23		
	4	15	7.36	1.24		0.41	0.34	0.21	
	5	26	7.21	1.68		0.22	0.28	0.08	0.09
	Total (*N*)	82	7.03	1.65	0.652				
Factor 6: Advocate	1	17	7.03	2.53					
	2	13	7.38	1.99		0.14			
	3	11	5.95	2.37		0.43	0.60		
	4	15	7.20	1.63		0.07	0.09	0.53	
	5	26	7.42	2.00		0.16	0.02	0.62	0.11
	Total (*N*)	82	7.10	2.11	0.396				

Results in Table 6 reveal a difference in midwives’ mean scores in five hospitals. Midwives in Hospitals 1 and 2 scored higher in Part I than those in other hospitals, which relates to cognitive aspects; midwives at both these hospitals are more knowledgeable on how cultural factors may influence maternal care, as evidenced by the mean score of Hospital 1 (7.92; *SD* = 1.51) and Hospital 2 (8.38; *SD* = 1.24), respectively. Midwives in Hospital 3 had a lower self-efficacy with a mean score of 5.78 (*SD* = 1.74) than all other hospitals. With the effect size, there is a large effect with Hospitals 4 and 5, with *d* scores of 0.80 and 0.85, respectively. Midwives in Hospital 2 had a practically significant association with midwives in Hospital 3 (*d* = 1.49), Hospital 4 (*d* = 0.78), and Hospital 5 (*d* = 0.83). Furthermore, midwives in Hospital 1 had a practically significant association with midwives in Hospitals 3, 4, and 5, ranging from a medium to large effect size, as evidenced by *d* scores of 1.23, 0.49, and 0.44, respectively. There is a practically significant association with knowledge and understanding cultural factors, which may affect maternal health care.

Part II of the questionnaire revealed that the midwives at Hospital 3 have a lower self-efficacy level than midwives in Hospitals 2 and 4 when interviewing patients of different cultural backgrounds, evidenced by an average mean score of 6.32 (*SD* = 1.98). Practically significant relationship is small between midwives in Hospitals 2 with 3 (*d* = 0.37) and Hospital 4 with 3 (*d* = 0.44), respectively.

The perception of self-efficacy levels of midwives in Part III (Factor 1), which deals with self-awareness, rated highest in Hospitals 4 and 5; these hospitals did not differ significantly, as midwives at these hospitals are more aware of their own cultural heritage and belief systems, biases, and limitations, as well as the differences within their cultural groups. However, Hospitals 1 and 2 have a medium practically significant relationship with Hospital 4 (*d* = 0.49) and Hospital 5 (*d* = 0.55).

Factor 2, focusing on the midwives’ awareness among patients of different cultural backgrounds, rated highest for Hospital 5, with a mean score of 7.71 (*SD* = 1.45). The midwives at Hospitals 1 and 3 were less aware of aspects such as insensitive and prejudicial treatment, differences in the perceived role of the nurse, traditional caring behaviors, professional caring behaviors (Items 57–62), as evidenced by a mean score of 6.95 (*SD* = 2.01) and 6.86 (*SD* = 1.89), respectively. There is a practically significant relationship between midwives at Hospitals 1 and 5 (*d* = 0.37), and midwives at Hospital 3 had a practical significant relationship with midwives at Hospital 5 (*d* = 0.*45*).

In Factor 3, named “Acceptance,” midwives at Hospital 4 were more confident in their willingness to accept differences and similarities between cultural groups and patient’s refusal of treatment based on beliefs, as evidenced by the highest mean score of 8.51 (*SD* = 1.08) among all five hospitals. Hospital 5 had the highest score of 7.67 (*SD* = 1.59), while Hospital 1 scored lowest with 7.16 (*SD* = 1.73). There was a medium effect size for Hospital 4, which implies a practically significant relationship with Hospitals 1 (*d* = 0.78) and 5 (*d* = 0.60), while with Hospital 3, the effect size was large (*d* = 0.84) for this part of the questionnaire. Furthermore, Hospital 4 had a practically significant relationship with Hospital 5, as evidenced by a medium effect size (*d* = 0.53).

Factor 4, named “Appreciate,” revealed that midwives at Hospital 4 have a higher confidence levels than other hospitals, with a mean score of 8.17 (*SD* = 1.25), respectively. The lowest score was from Hospital 1, with a mean score of 7.41 (*SD* = 1.87). However, there was a practical significant relationship between Hospitals 1 and 4 (*d* = 0.41). Another practical significant relationship occurred with Hospitals 4 and 5, as evidenced by an effect size of 0.37. This part addresses aspects such as interaction with people of different cultures, cultural sensitivity and awareness, culture-specific nursing care role of the family in providing health care, and the patient’s worldview.

Factor 5, named “Recognize,” included aspects such as inadequacies in the SA health care system and the importance of roles in health care practices, among others. The midwives at Hospital 2 had the lowest confidence level, evidenced by a mean of 6.50 (*SD* = 2.53). Hospital 4 scored higher than Hospital 2, with a mean of 7.36 (*SD* = 1.24). Overall, the hospitals’ level of confidence for this factor was moderate, with a mean score of 7.03 (*SD* = 1.65). As per the previous aspect of the questionnaire, there was evidence of a practical significant relationship between Hospitals 1 and 4 (*d* = 0.41).

The last factor, named “Advocate,” is related to the important role of the midwives when providing maternal care to patients. Health professionals should pursue justice and advocate for better care for vulnerable and disadvantaged patients (South African Nursing Council [SANC], 2013). Midwives at Hospital 3 are less confident in advocating for patients regarding their decisions based on cultural beliefs and culture-specific care, as evidenced by the lowest mean score of 5.95 (*SD* = 2.37). There is a practically significant relationship between Hospitals 1 and 3 (*d* = 0.43), while Hospital 2 had a moderate practically significant relationship with Hospital 3 (*d* = 0.60). Furthermore, medium effect size practically significant relationship was evident between Hospitals 3, 4 (*d* = 0.53), and 5 (*d* = 0.62).

### Correlation Between the Items on the Subscales

Table 7 depicts the correlation between items on the TSET questionnaire. There were no negative correlations reported between the subscales.

**Table 7.** Correlations Between Items on the Subscales.

Items		Part I (Knowledge and understanding)	Part II (Interviewing)	Part III Factor 1: Self-awareness	Factor 2: Patients’ awareness	Factor 3: Acceptance	Factor 4: Appreciate	Factor 5: Recognize	Factor 6: Advocate
Part I (Knowledge and understanding)	Correlation coefficient	1.000	.615**	.234*	.512**	.441**	.542**	.536**	.538**
	Sig. (two-tailed)		0.000	0.034	0.000	0.000	0.000	0.000	0.000
	*N*	82	82	82	82	82	82	82	82
Part II (Interviewing)	Correlation coefficient	.615**	1.000	.307**	.698**	.604**	.666**	.698**	.555**
	Sig. (two-tailed)	0.000		0.005	0.000	0.000	0.000	0.000	0.000
	*N*	82	82	82	82	82	82	82	82
Part III Factor 1: Self-awareness	Correlation coefficient	.234*	.307**	1.000	.565**	.497**	.466**	.375**	.354**
	Sig. (two-tailed)	0.034	0.005		0.000	0.000	0.000	0.001	0.001
	*N*	82	82	82	82	82	82	82	82
Factor 2: Patients’ awareness	Correlation coefficient	.512**	.698**	.565**	1.000	.719**	.684**	.709**	.526**
	Sig. (two-tailed)	0.000	0.000	0.000		0.000	0.000	0.000	0.000
	*N*	82	82	82	82	82	82	82	82
Factor 3: Acceptance	Correlation coefficient	.441**	.604**	.497**	.719**	1.000	.710**	.707**	.616**
	Sig. (two-tailed)	0.000	0.000	0.000	0.000		0.000	0.000	0.000
	*N*	82	82	82	82	82	82	82	82
Factor 4: Appreciate	Correlation coefficient	.542**	.666**	.466**	.684**	.710**	1.000	.720**	.555**
	Sig. (two-tailed)	0.000	0.000	0.000	0.000	0.000		0.000	0.000
	*N*	82	82	82	82	82	82	82	82
Factor 5: Recognize	Correlation coefficient	.536**	.698**	.375**	.709**	.707**	.720**	1.000	.582**
	Sig. (two-tailed)	0.000	0.000	0.001	0.000	0.000	0.000		0.000
	*N*	82	82	82	82	82	82	82	82
Factor 6: Advocate	Correlation coefficient	.538**	.555**	.354**	.526**	.616**	.555**	.582**	1.000
	Sig. (two-tailed)	0.000	0.000	0.001	0.000	0.000	0.000	0.000	
	*N*	82	82	82	82	82	82	82	82

* Correlation is significant at the .05 level (two-tailed).

** Correlations significant at the .01 level (two-tailed).

Part I (Cognitive subscale), with items that relate to “knowledge and understanding of different cultural factors,” correlates positively with Part II (Practical subscale), which relates to the “perception of the midwives” confidence in interviewing patients of different cultural backgrounds; correlation coefficient was .615. Part I also correlates positively with all the factors of Part III (Affective subscale), as evidenced again between Part II and the factors of Part III. The highest correlation is between “midwives” awareness of the patients of different cultural backgrounds and recognition of other factors relating to inadequacies in the SA health care system, as well as the importance of home remedies and indigenous medicine, among others; this is evidenced by a correlation coefficient of .698. Overall, the correlations range from medium to large between the factors. The smallest correlation was evidenced between Part I and Part II, with Factor 1 of Part III which relates to the midwives’ self-awareness regarding their own cultural heritage and belief systems, biases, and limitation, as well as differences within their cultural group; the correlation coefficients are .234 and .307, respectively.

This result tells us that when the midwives know about the patients’ different cultures and how those affect their care, their perception of their confidence in interviewing patients of different cultures increases. Their confidence levels will also increase regarding their awareness among patients of different cultural backgrounds, their willingness to accept the differences, and for them to appreciate such differences. Finally, this will increase their confidence level in recognizing other factors relating to inadequacies in the SA health care system the importance of home remedies and indigenous medicine, among others. Midwives will end up with increased confidence in advocating for patients’ decisions based on cultural beliefs as well as culture-specific care when they know about all the cultural factors, which may affect their nursing care. The highlighted column in the table represents a positive intercorrelation between the factors and all the subscales.

## Reliability and Validity

The reliability of the instrument explains the consistency of the measures obtained and results in less measurement error (Gray et al., 2017). Previously, the developers found the reliability and validity of the TSET questionnaire were within the range of .92 to .98 (Jeffreys & Dogan, 2010). The statistician who assisted with the data analysis also measured the reliability in the current study Cronbach’s alpha ranged between .76 and .98, indicating the instrument’s reliability and validity. Cronbach’s alpha coefficients range from .00, indicating no internal consistency, to 1.00, indicating perfect internal reliability, with no measurement error.

Validity of the TSET questionnaire was tested. The validity of the survey instrument is indicative of the extent to which it is capable of measuring the construct being measured (Gray et al., 2017), which is the cultural competence of the midwives in the current study. Exploratory factor analysis determined the validity of the TSET questionnaire and the relationship between various items on the instrument. During factor analysis, the variables were sorted into categories according to how closely related they were, the grouping of such variables evidenced this was evidenced by the grouping of such variables into a factor (Gray et al., 2017).

## Discussion

This study was unique in that it used the TSET questionnaire to assess the transcultural self-efficacy (TSE) of midwives in five different hospitals within NWP and placed emphasis on the practical significance of the findings rather than the statistical significance. To the best knowledge of the authors, this is the first study to assess the cultural competence of midwives in SA using TSET. Internationally, most of the studies of cultural competence using TSET were conducted with undergraduate nursing students (Asurakkody, 2019; Halter et al., 2015; Jeffreys & Dogan, 2012; Jin & Cleofas, 2018; Li et al., 2016; Malliarou et al., 2017). However, Li et al. (2016) conducted a study in three hospitals in China with registered nurses, while Jin and Cleofas (2018) conducted their study in four hospitals with staff nurses in the Philippines. According to Jeffreys and Dogan (2012), individuals with resilient TSE perceptions will be motivated and committed to learning and performing culturally competent nursing care.

This study explored the relationship between the demographic data and the transcultural self-efficacy levels of the midwives, concerning age, qualification, and home language. Halter et al. (2015) found no significant relationship with most demographic variables except academic level and prior health care experience. In their study, Jin and Cleofas (2018) found no positive relationship between the age of nurses and their clinical experiences in the hospital. Conversely, Li et al. (2016) concluded that the TSE of nurses in China was higher for older, more experienced nurses with higher professional titles and incomes.

A study by Asurakkody (2019, p. 3) with nursing students revealed that demographic factors such as geographic location, age, gender, and relationship with multicultural groups were significantly associated with TSE. In this current study, there was no significant relationship with the participants’ home language. However, some concurrence was evidenced in Asurakkody’s study with one variable, age. This study also found that the age of the midwives has a positive correlation with the factors in the TSET questionnaire, yet qualification does not have a positive correlation. It is evident that although only a limited number of demographic variables were used, there is some similarity with previous studies on the association between TSET factors and age (Asurakkody, 2019; Li et al., 2016). An increase in the age of midwives increases their confidence levels on items in the cognitive subscale of the tool as well as the affective subscale, namely, patients’ awareness and advocacy.

Lim et al. (2004) found that students had a lower perception of self-efficacy in the cognitive subscale. Contrary to their findings, Malliarou et al. (2017) found that the military nurses in Greece had the highest score in knowledge questions, mostly related to hygiene and maintenance of health. The findings of the current study reveal that the mean score for knowledge and understanding of cultural factors is moderate; however, the authors emphasize that the difference is that these midwives had no exposure to any transcultural training as were the Greek nurses or the students in the study by Lim et al. (2004). Findings about the midwives’ moderate levels of self-efficacy are supported by the speculation of Li et al. (2016) that although the midwives did not have prior training, their years of experience in the nursing field can lead to more confidence when dealing with patients from different cultures. Jin and Cleofas (2018) also support the notion that nurses’ experience gained over the years influences their cognitive domain.

It is essential for the midwife to interview patients about cultural values and beliefs, which assists them in developing a culture-specific/culturally competent or culturally congruent care plan (Jeffreys & Dogan, 2010). Therefore, it is a concerning finding that the midwives’ confidence in interviewing patients from different cultural backgrounds on factors such as acculturation (item 38) and world view (item 39) was the lowest. This finding is consistent with the finding on the military nurses by Malliarou et al. (2017), even though they had higher scores on importance of verbal or nonverbal communication. Conversely, Lim et al. (2004) found that nursing students had higher self-efficacy in the practical and affective subscales. The findings indicated that midwives need training or education on transcultural skills.

This study reported the effect size, calculated according to Cohen as *d* (Ellis & Steyn, 2003; Sink & Stroh, 2006). The belief is that this discussion of effect sizes will provide the practical importance of the findings of the five hospitals about cultural competence of midwives. Although the number of respondents across the hospitals was not equal, Hojat and Xu (2004) emphasized that the effect size is an index that quantifies the degree to which the study findings are considered necessary, regardless of the sample size. The authors found no reports of effect size on similar studies of cultural competence using the TSET questionnaire.

About Part I of the TSET, there is a practically significant relationship between midwives in Hospitals 3, 4, and 5. This means midwives at these hospitals have more confidence in their knowledge and understanding of cultural factors, which may affect maternal care, compared with the midwives in Hospitals 1 and 2. Part II of the questionnaire revealed a small, practically significant relationship between midwives in Hospitals 2 and 3 with midwives in Hospital 4. Furthermore, in Part III, there is a small and practically significant relationship between midwives in Hospitals 1 and 4 and midwives in Hospitals 2 and 4. There is evidence of a medium size effect between midwives in Hospitals 1 and 2, with midwives in Hospitals 4 and 5. This finding relates to the confidence of midwives regarding their self-awareness (Factor 1).

Part III further showed small practical significance between midwives in Hospital 1 with Hospital 5 and midwives in Hospital 3 with Hospital 5. Midwives at Hospital 4 are more willing to accept differences and similarities between their patients (Factor 3), as evidenced by a large effect size with Hospital 3. A small, practically significant relationship exists between midwives in Hospital 1 with 4 and between Hospital 4 with 5 on the midwives’ appreciating interaction with people of different cultures, cultural sensitivity and awareness, culture-specific nursing care, the role of the family in providing care, and patient’s worldview (Factor 4). Still on the affective subscale, concerning recognizing different aspects among patients of different cultural backgrounds (Factor 5), the only notable result was a small and practically significant relationship between midwives in Hospitals 1 and 4.

The last factor on the subscale refers to the midwives’ confidence in advocating for patients’ decisions based on cultural beliefs and culture-specific care (Factor 6). There is a small to medium practical significance between midwives in hospitals, with the relationship between Hospital 3 with Hospital 5 having a larger effect size. This means midwives at these hospitals have more confidence in patient advocacy.

## Conclusion

This study was unique as it measured the self-reported levels of midwives in the NWP of SA using TSET. All the subscales in the TSET questionnaire are essential in the development of cultural competence as well as for the midwives to be able to provide culturally congruent maternal care (Jeffreys & Dogan, 2010). Therefore, a positive correlation was noted between all the factors in the TSET questionnaire. This finding should have been noted in the literature on studies of cultural competence that used the TSET questionnaire. A positive correlation between these factors tells us that if the midwives in NWP have knowledge and understanding of cultural factors, which may affect their care, then all the other factors will increase, ultimately rendering them culturally competent. It is recommended that midwives receive training to improve their cultural competence levels. Further research can also be conducted, including developing a cultural competence tool within the South African context.

## Limitations

The sample size only included (*N* = 104, *n* = 78) midwives, due to midwife shortages in the province. Due to the sample size, the study is not generalizable to the entire SA, but could, however, be used as a guide for other provinces. Maternity units are generally hectic and understaffed, and the midwives did not find a suitable time to complete the questionnaires at work. The few midwives who took questionnaires home did not return them to the hospital.
